# Effect of two prostaglandin injections on days 5 and 6 in a timed AI protocol after estrus expression on pregnancy outcomes in dairy cows during cold or hot seasons of the year

**DOI:** 10.5194/aab-62-161-2019

**Published:** 2019-04-10

**Authors:** Mufeed A. Alnimer, Mohamed A. Abedal-Majed, Ahmad I. Shamoun

**Affiliations:** Department of Animal Production, School of Agriculture, University of Jordan, Amman 11942, Jordan Amman

## Abstract

The objective of this study was to test whether prostaglandin (PG) injection on day 30 postpartum (pp) and detection of estrus can affect the efficacy of injecting PG on days 5 and 6 in the timed artificial insemination (TAI) protocol on pregnancy rate in a large dairy herd in hot or cold seasons. Out of 2235 cows, 1998 received an injection of PG at 30±3 d pp and estrus was observed. Cows that displayed estrus during the estrous observation period after PG injection were classified as estrus (E), while those that did not show estrus were classified as nonestrus (NE). Cows in each group were assigned to two treatments: CO-72 (control treatment) (ECO-72 and NECO-72) (day 44 GnRH, day 51 PGF${}_{2\alpha }$, day 54 GnRH+TAI) or PG–PG (EPG–PG and NEPG–PG) (day 44 GnRH, day 49 PGF${}_{2\alpha }$, day 50 PGF${}_{2\alpha }$, day 52 GnRH+TAI). Pregnancy was diagnosed on days 33 and 47 after artificial insemination (AI). The proportion of cows in estrus on the day of TAI was higher (P≤0.05) for cows that received two PG than for cows that received one PG. Pregnancies per AI (P/AI) on days 33 and 47 for cows inseminated during and after a voluntary waiting period in the NEPG–PG treatment had higher rates than for cows in the EPG–PG, ECO-72 and NECO-72 treatments. Moreover, P/AI were significantly (P≤0.05) affected by parity. Primiparous had higher P/AI (37.0 %) than multiparous cows (31.6 %). Cows inseminated in cold months had higher P/AI and reduced PL (35.6 % and 20.8 %) than cows inseminated in hot months (29.1 % and 30.6 %, respectively). In conclusion, treatments with PG on days 5 and 6 after the first GnRH injection increased P/AI. Estrus detection before the beginning of TAI protocol did not affect fertility. To maximize P/AI cows exhibiting heat at any time during the synchronization protocol should be inseminated.

## 1 Introduction

The reproductive management of dairy cows around the world is declining as greater selection pressure for increased milk production is applied. Conception rates to the first insemination have dropped in the last 16 years (Washburn et al., 2002). Our goal is to have the highest rate of pregnancy per AI at first insemination postpartum in order to reduce the calving interval and open days, which are inversely proportional to milk production.

Timed AI protocols were developed to synchronize three basic events in the estrous cycle of the dairy cow: follicular wave emergence, corpus luteum (CL) regression and ovulation, and all three events can be synchronized if the TAI protocol is initiated between days 5 and 12 of the estrous cycle, which requires presynchronization (Vasconcelos et al., 1999; Moreira et al., 2001; Navanukraw et al., 2004). Ovulation at the first GnRH injection is a key determinant for success in TAI protocols and is correlated with an increase in fertility. However, previous studies have shown an increased concentration of progesterone (P4) near the time of AI, which means that the CL did not regress completely, and this led to suboptimal P/AI (Pursley et al., 1997; Moreira et al., 2001; Souza et al., 2008). Therefore, injection of another dose of PG was used to attempt to overcome the problem of incomplete regression of CL.

Previously, Brusveen et al. (2009) added another PG to the original Ovsynch protocol 24 h after the initial one and found that it led to an increase in luteolysis and synchronization accuracy but gained no improvement in P/AI. These results were similar to those of Wiltbank et al. (2015), who added a PG dose to the original Ovsynch and Double Ovsynch, except that Wiltbank et al. (2015) found a tendency for increasing P/AI for the two PG-treated cows. Restriction of the follicle dominance to 5–6 d was reported by Cerri et al. (2009) to improve embryo quality. Increasing the follicular dominance by as few as 1.5 d can compromise embryonic survival (Cerri et al., 2009). This idea was used by Santos et al. (2010) and Ribeiro et al. (2012) to shift back the PG from day 7 to day 5 and to add another dose on day 6 in the Cosynch 72 protocol to avoid the problem of incomplete regression of CL, particularly in presynchronized cows which have a high ovulation rate in response to the first GnRH. The results of both studies indicated an overall improvement in fertility, including lower P4 concentrations and higher CL regression at TAI, a smaller diameter of the ovulatory follicle, higher P/AI and the tendency for an increasing synchronization rate for cows that received two PG injections on days 5 and 6 of the Cosynch 72 protocol. However, applying two PG injections is labor intensive for dairy farm managers since it requires another cow handling session. Therefore, adjustment of the PG injection to once on day 6 was attempted by Yilmazbas-Mecitoglu et al. (2013) and Stevenson et al. (2014), but the results of fertility response were not encouraging, even though Stevenson et al. (2014) used a doubled dose of PG. The objective of this study was to test whether PG injection on day 30 pp and detection of estrus can affect the efficacy of injecting PG on days 5 and 6 in the TAI protocol compared to the Cosynch protocol in a large dairy herd in hot or cold seasons.

## 2 Materials and methods

All procedures were approved by the Scientific Research Ethics Committee at the University of Jordan, Amman.

### 2.1 Cows, housing and management

Lactating Holstein Friesian dairy cows were housed in free-stall barns provided with shade on a commercial dairy farm located in the Al-Khalidia area of the northern part of Jordan at 32${}^{\circ }$2${}^{\prime }$ N, 35${}^{\circ }$51${}^{\prime }$ E during the period between January 2014 and June 2015. Cows were milked three times daily at 8 h intervals with an average milk yield of around 8000 kg per lactation. Cows were fed a total mixed ration (TMR) of 40 % forage (corn silage and alfalfa hay) and 60 % concentrate (corn, barley, wheat bran, soybean meal and commercial concentrate for lactation with trace minerals and vitamins) containing 1.8 Mcal net energy of lactation (NE${}_{L}$) kg${}^{-1}$, 19 % crude protein (CP) (dry matter basis) and the feed was changed according to National Research Council (NRC) recommendations (2001). Cows had free access to fresh water. Meteorological data consisting of daily maximum temperatures and relative humidity were obtained from the Official National Station in the Alkhaldia area 2 km away from the farm. The mean maximum temperature is 36.5±1.0 ${}^{\circ }$C and 22.5±0.6 ${}^{\circ }$C, minimum temperature is 17.0±0.3 ${}^{\circ }$C and 10.0±0.2 ${}^{\circ }$C and relative humidity is 56.8 % and 64.4 % during the experimental period for hot (June to September) and cold (October to May) months.

### 2.2 Experimental design

**Figure 1:**
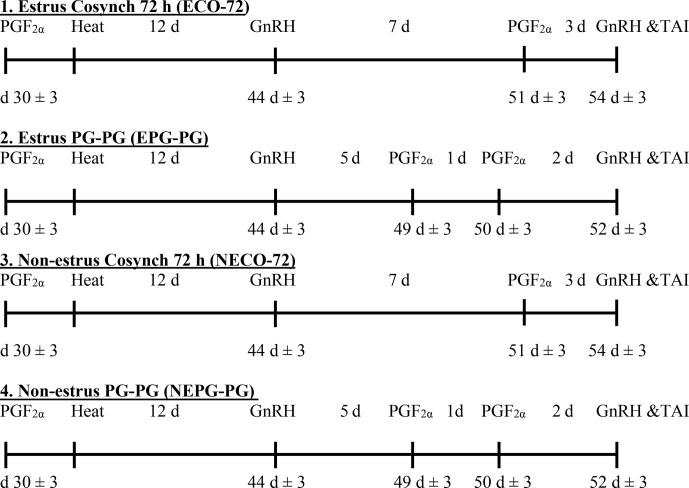
Schematic diagram of the different hormonal treatments of the lactating cows in the study.

A total of 2312 lactating Holstein Friesian dairy cows were subjected to an estrus detection protocol starting on Day 25 pp. The program included an ALPRO system with an activity meter (Delaval International AB, Tumba, Sweden) fitted to the neck of every cow to detect and record the activity exhibited by the cow when she approached heat and transmitted data every hour to the computer. In addition, standing heat was confirmed by visual observation. Seventy-seven cows were excluded from the study due to disease and culling for udders and structure, while 2235 cows (primiparous, n=706; and multiparous, n=1529) were injected 25 mg PG i.m. (Lutalyse; Pharmacia & Upjohn S.A., Puurs, Belgium) on Day 30±3 pp and subjected to an estrus detection protocol as described above for 1 week. Cows that showed estrus during the estrus observation periods after PG injection were classified as estrus cows (E; n=830), while those that did not show estrus were classified as nonestrus cows (NE; n=1405). Cows from each estrus category were assigned to two treatments without using any progesterone supplements: CO-72 (ECO-72; n=430 and NECO-72; n=576), which received an injection of 10 µg GnRH agonist (Buserelin, Receptal^®^, Hoechst Roussel Vet GmbH) on Day 44±3, PGF${}_{2\alpha }$7 d later and GnRH with TAI 72 h after PGF${}_{2\alpha }$; and PG–PG (EPG–PG; n=400 and NEPG–PG; n=829), which received an injection of GnRH on Day 44±3 Day 4, PGF${}_{2\alpha }$5 and 6 d later and GnRH with TAI 48 h after the second PGF${}_{2\alpha }$ (Fig. 1). An experienced AI technician performed insemination with commercially available frozen semen of proven fertility (ABS Global, Inc., Deforest, Wisconsin, USA). Semen source was randomized across the treatments. In addition, a routine examination for semen was conducted every 2 months in order to be sure that there was no change in the semen quality.

Cows were examined for pregnancy on Day 33±3 of either TAI or AI, using an ultrasound (scanner 100 Vet; Pie Medical, Maastricht, the Netherlands) with a 7.5 MHz probe. Pregnancy was determined by visualization of an embryonic vesicle with a heartbeat as described (Pierson and Ginther, 1984). Pregnancy status was confirmed by rectal palpation on Day 47±3 after insemination. Pregnancy losses were calculated as the difference between cows pregnant at the first examination and cows not pregnant at the second examination.

Average milk production between days 30 and 90 pp was 28.4±0.5 kg d${}^{-1}$, and the average in the first 120 d pp was similar (P≥0.05) for cows in the EPG–PG, NEPG–PG, ECO-72 and NECO-72 treatments (27.9±0.5, 28.1±0.4, 28.6±0.5 and 28.5±0.4 kg d${}^{-1}$, respectively) and did not affect pregnancy rate. Likewise, mean lactation numbers for cows in the EPG–PG (2.29±1.5), NEPG–PG (2.44±1.3), ECO-72 (2.29±1.3) and NECO-72 (2.28±1.3) treatments were not significantly different.

### 2.3 Statistical analysis

Statistical analysis was conducted using SAS (Version 9; SAS Institute, 2002). Data were evaluated using PROC LOGISTIC, PROC GLM and PROC FREQ in SAS. In total, 1998 cows with complete hormonal protocol were included in the final statistical analysis. The model included the treatment effects of estrus expression (estrus vs. nonestrus), treatment (EPG–PG vs. ECO-72 vs. NECO-72 vs. NEPG–PG), parity (primiparous vs. multiparous), season (hot vs. cold) and their interactions. To carry out the statistical analyses, data were coded as 1 (pregnant) or 0 (not pregnant) per AI on days 33 and 47 after TAI. Chi-square analysis using the PROC FREQ procedure was used to test independent variables among treatments groups, between parity, season and P/AI (days 33 and 47), and pregnancy losses among treatment groups. Estrus detection rate before the last GnRH administration and within 7 d after TAI in the four treatments was tested by a chi-square test using the FREQ procedure of SAS (2002). Continuous data from calving to voluntary waiting period (VWP) days were analyzed using the general linear models procedure of SAS (2002). The effects of the average milk yield for the first 4 months and environmental data during experimental period on treatments and pregnancy rates were estimated. Least square means for significant effects were compared at P≤0.05 using a t test.

## 3 Results

### 3.1 Distribution of cows according to voluntary waiting period (VWP) 

Table 1 shows the distribution of cows in the four treatments according to VWP. Cows that showed premature estrus (n=237) were inseminated before the last GnRH administration (before VWP) and were excluded from the study analysis because these cows did not complete the hormonal protocols. The majority (n=1783) of cows in the EPG–PG, NEPG–PG, ECO-72 and NECO-72 treatments that completed the protocols were artificially inseminated during the VWP on days 52.6±0.4, 52.6±0.7, 53.3±0.5 and 53.7±0.5 pp, respectively. Cows that returned to estrus after the VWP (n=215) were re-inseminated without exclusion from the study. Therefore, the 1998 cows that completed the hormonal protocols were available for analysis.

**Table 1:** Estrus detection rate for the four treatments according to the voluntary waiting period (VWP).

	Treatment${}^{1}$			
Estrus detection	EPG–PG	NEPG–PG	ECO-72	NECO-72
rate${}^{2}$ (%)	(n=400)	(n=829)	(n=430)	(n=576)
	(n) %	(n) %	(n) %	(n) %
Before VWP${}^{3}$	(22) 5.5${}^{c}$	(78) 9.4${}^{b}$	(64) 14.9${}^{a}$	(73) 12.7${}^{\mathrm{ab}}$
During VWP${}^{4}$	(330)82.5${}^{a}$	(662) 79.9${}^{b}$	(335) 77.9${}^{c}$	(456) 79.2${}^{\mathrm{bc}}$
After VWP${}^{5}$	(48) 12${}^{a}$	(89) 10.7${}^{b}$	(31) 7.2${}^{c}$	(47) 8.2${}^{c}$

${}^{1}$ Cows showing estrus during the postpartum period received the CO-72 (EPG–PG) (GnRH+PGF${}_{2\alpha 1}+$PGF${}_{2\alpha 2}+$GnRH+TAI 48 h after PGF${}_{2\alpha 2}$) or ECO-72 (GnRH+PGF${}_{2\alpha }+$GnRH+TAI 72 h after PGF${}_{2\alpha }$). Cows that did not show estrus during the postpartum period received the CO-72 (NEPG–PG) (GnRH+PGF${}_{2\alpha 1}+$PGF${}_{2\alpha 2}+$GnRH+TAI 48 h after PGF${}_{2\alpha 2}$) or NECO-72 (GnRH+PGF${}_{2\alpha }+$GnRH & TAI 72 h after PGF${}_{2\alpha }$). ${}^{2}$ By ALPRO^™^ system and visual observation. ${}^{3}$ Cows exhibited estrus and were inseminated before the last GnRH in the four treatments. ${}^{4}$ Cows were inseminated during the VWP (48 or 72 h after the last PGF${}_{2\alpha }$). ${}^{5}$ Cows exhibited estrus and were re-inseminated within 7 d after the end of the protocol in the four treatments. ${}^{a,b,c}$ Percentages among treatments with different superscripts differ (P≤0.05).

The proportion of cows in estrus based on the ALPRO system and visual observation on the day of TAI was higher (P≤0.05) for cows that received two PG {EPG–PG and NEPG–PG (992/1229) 80.7 %} than for cows that received one PG {ECO-72 and NECO-72 (791/1006) 78.6 %}. This pattern is reflected in multiparous cows, where the proportion of cows in estrus on the day of TAI was higher (P≤0.05) for cows that received two PG (679/817, 83.1 %) than for cows that received one PG (558/712, 78.4 %) treatment. In contrast, in primiparous cows, the proportion of cows in estrus on the day of TAI was lower (P≤0.05) for cows that received two PG (313/412, 76.5 %) than for cows that received one PG (233/294, 79.3 %) treatment. Moreover, P/AI were affected by estrus expression; nonestrus groups (36.5 %) had more pregnancies than estrus groups (27.8 %).

### 3.2 Treatment, parity and season effect on pregnancies per AI (P/AI)

According to Table 2, the logistic regression revealed that P/AI on Day 33 were significantly associated with treatment (P=0.001) and parity (P=0.004) but not season. In contrast, P/AI on Day 47 were significantly associated with treatment (P=0.001), parity (P=0.028) and season (P=0.033) (Table 3). Pregnancies per AI on days 33 and 47 for cows inseminated during and after VWP in the NEPG–PG (59.7 % and 45.1 %) treatment were higher (P≤0.05) than for cows in the EPG–PG (33.6 % and 28.8 %), ECO-72 (36.9 % and 26.8 %) and NECO-72 (33.0 % and 23.7 %) treatments. Moreover, P/AI were higher on days 33 and 47 in primiparous (49.8 % and 37.0 %) cows (P≤0.05) than in multiparous (41.2 % and 31.6 %) cows. In addition, P/AI on Day 47 was higher (P≤0.05) in the cold season (35.6 %) than in the hot (29.1 %) season.

**Table 2:** Odds ratios of the variables included in the final logistic regression model for factors affecting pregnancies per AI on Day 33±3 post-TAI.

Factors	Pregnancies per AI % (n)	Odds ratio	95 % CI	P value
Treatment${}^{1}$				
EPG–PG	33.6${}^{b}$ (127/378)	Reference		0.001
NEPG–PG	59.7${}^{a}$ (448/751)	2.95	2.32–3.74	
ECO-72	36.9${}^{b}$ (135/366)	1.02	0.77–1.37	
NECO-72	33.0${}^{b}$ (166/503)	1.19	0.90–1.59	
Parity				
Primiparous	49.8${}^{a}$ (311/625)	Reference		0.004
Multiparous	41.2${}^{b}$ (565/1373)	1.33	1.09–1.62	
Season				
Hot	41.9 (294/702)	Reference		0.921
Cold	44.9 (582/1296)	0.95	0.78–1.16	

CI is confidence interval. ${}^{1}$ Cows showing estrus during the postpartum period received the CO-72 (EPG–PG) (GnRH+PGF${}_{2\alpha 1}+$PGF${}_{2\alpha 2}+$GnRH+TAI 48 h after PGF${}_{2\alpha 2}$) or ECO-72 (GnRH+PGF${}_{2\alpha }+$GnRH+TAI 72 h after PGF${}_{2\alpha }$). Cows that did not show estrus during the postpartum period received the CO-72 (NEPG–PG) (GnRH+PGF${}_{2\alpha 1}+$PGF${}_{2\alpha 2}+$GnRH+TAI 48 h after PGF${}_{2\alpha 2}$) or NECO-72 (GnRH+PGF${}_{2\alpha }+$GnRH & TAI 72 h after PGF${}_{2\alpha }$). ${}^{a,b}$ Percentages among treatments and parity with different superscripts differ (P≤0.05).

**Table 3:** Odds ratios of the variables included in the final logistic regression model for factors affecting pregnancies per AI on Day 47±3 post-TAI.

Factors	Pregnancies per AI % (n)	Odds ratio	95 % CI	P value
Treatment${}^{1}$				
EPG–PG	28.8${}^{b}$ (109/378)	Reference		0.001
NEPG–PG	45.1${}^{a}$ (339/751)	2.56	1.99–3.30	
ECO-72	26.8${}^{b}$ (98/366)	1.25	0.92–1.69	
NECO-72	23.7${}^{b}$ (119/503)	1.19	0.88–1.63	
Parity				
Primiparous	37.0${}^{a}$ (231/625)	Reference		0.028
Multiparous	31.6${}^{b}$ (434/1373)	1.19	0.97–1.45	
Season				
Hot	29.1${}^{b}$ (204/702)	Reference		0.033
Cold	35.6${}^{a}$ (582/1296)	0.80	0.65–0.99	

CI is confidence interval. ${}^{1}$ Cows showing estrus during the postpartum period received the CO-72 (EPG–PG): (GnRH+PGF${}_{2\alpha 1}+$PGF${}_{2\alpha 2}+$GnRH+TAI 48 h after PGF${}_{2\alpha 2}$) or ECO-72 (GnRH+PGF${}_{2\alpha }+$GnRH+TAI 72 h after PGF${}_{2\alpha }$). Cows that did not show estrus during the postpartum period received the CO-72 (NEPG–PG): (GnRH+PGF${}_{2\alpha 1}+$PGF${}_{2\alpha 2}+$GnRH+TAI 48 h after PGF${}_{2\alpha 2}$) or NECO-72 (GnRH+PGF${}_{2\alpha }+$GnRH & TAI 72 h after PGF${}_{2\alpha }$). ${}^{a,b}$ Percentages among treatments, parity and season with different superscripts differ (P≤0.05).

Pregnancy losses (PLs) between days 33 and 47 after TAI are displayed in Table 4. Logistic regression revealed that treatment- and season-influenced PL (P=0.086 and P=0.034, respectively), but no significant association was detected for parity. Pregnancy losses for cows in the EPG–PG (14.2 %) treatment tended (P≤0.1) to be lower than for cows in the NEPG–PG (24.3 %), ECO-72 (27.4 %) and NECO-72 (28.3 %) treatments, whereas pregnancy losses were similar between primiparous (25.7 %) and multiparous (23.2 %) cows (Table 4). In the hot months of June to September, irrespective of parities, cows had higher PLs (30.6 %) than during the remaining months (20.8 %).

**Table 4:** Odds ratios of the variables included in the final logistic regression model for factors affecting pregnancy losses between days 33±3 and 47±3 post-TAI.

Factors	Pregnancy losses	Odds ratio	95 % CI	P value
Treatment${}^{1}$				
EPG–PG	14.2${}^{c}$ (18/127)	Reference		0.086
NEPG–PG	24.3${}^{d}$ (109/448)	0.89	0.59–1.34	
ECO-72	27.4${}^{d}$ (37/135) 0.48	0.26–0.88		
NECO-72	28.3${}^{d}$ (47/166)	0.93	0.56–1.55	
Parity				
Primiparous	25.7 (80/311)	Reference		0.332
Multiparous	23.2 (131/565)	1.17	0.85–1.62	
Season				
Hot	30.6${}^{a}$ (90/294)	Reference		0.034
Cold	20.8${}^{b}$ (121/582)	1.57	1.13–2.19	

CI is confidence interval. ${}^{1}$ Cows showing estrus during the postpartum period received the CO-72 (EPG–PG): (GnRH+PGF${}_{2\alpha 1}+$PGF${}_{2\alpha 2}+$GnRH+TAI 48 h after PGF${}_{2\alpha 2}$) or ECO-72 (GnRH+PGF${}_{2\alpha }+$GnRH+TAI 72 h after PGF${}_{2\alpha }$). Cows that did not show estrus during the postpartum period received the CO-72 (NEPG–PG): (GnRH+PGF${}_{2\alpha 1}+$PGF${}_{2\alpha 2}+$GnRH+TAI 48 h after PGF${}_{2\alpha 2}$) or NECO-72 (GnRH+PGF${}_{2\alpha }+$GnRH & TAI 72 h after PGF${}_{2\alpha }$). ${}^{a,b}$ Percentages between season groups with different superscripts differ (P≤0.05). ${}^{c,d}$ Percentages among treatments with different superscripts tended to be differ (P≤0.1).

Regardless of the estrus expression before the initiation of the protocols, overall cows treated with PG on days 5 and 6 in the TAI protocol had significantly higher (P≤0.05) P/AI on Day 47 than cows in the Cosynch protocol (39.7 vs. 24.9 %); this was true in primiparous cows (42.9 vs. 28.4 %), in multiparous cows (38.1 vs. 23.6 %), in the hot season (36.8 vs. 23.3 %) and in the cold season (40.8 vs. 26.4 %) respectively. In addition, PLs between days 33 and 47 after TAI for cows treated with PG on days 5 and 6 in the TAI protocol were significantly lower (P≤0.05) than for cows in the Cosynch protocol (22.1 vs. 27.9 %), in multiparous cows (20.4 vs. 28.2 %), and between cold and hot seasons (20.8 vs. 30.6 %) but not between primiparous and multiparous cows (25.7 vs. 23.2 %) or within primiparous cows (25.0 vs. 27.3 %).

### 3.3 Interaction effect of treatment by parity on pregnancies per AI (P/AI)

Pregnancy outcomes according to the interaction of treatment and parity are shown in Table 5. Pregnancy rates on days 33 and 47 post-TAI were detected between the treatment and parity. Within primiparous cows, P/AI in cows receiving NEPG–PG protocol were higher (P≤0.05) than those in the cows receiving EPG–PG, ECO-72 and NECO-72 protocols on Day 33, while on Day 47 cows receiving the NEPG–PG protocol had higher rates (P≤0.05) than cows receiving ECO-72 and NECO-72 protocols but had similar rates to those cows receiving the EPG–PG protocol. However, within multiparous cows, P/AI were higher (P≤0.05) on days 33 and 47 for cows in NEPG–PG than for cows in the EPG–PG, ECO-72 and NECO-72 treatments. No interaction effect of treatment–parity was detected on P/AI on days 33 and 47 (P=0.55, P=0.25, respectively).

**Table 5:** Pregnancies per AI and pregnancy losses for cows based on treatment–parity interaction.

Parameter	Primiparous				Multiparous			
	Treatments${}^{1}$							
	EPG–PG	NEPG–PG	ECO-72	NECO-72	EPG–PG	NEPG–PG	ECO-72	NECO-72
	(n=102)${}^{2}$	(n=269)	(n=103)	(n=151)	(n=276)	(n=482)	(n=263)	(n=352)
Pregnancies per AI								
On Day 33±3	(43) 42.2${}^{b}$	(169) 62.8${}^{a}$	(40) 38.8${}^{b}$	(59) 39.1${}^{b}$	(84) 30.4${}^{b}$	(279) 57.9${}^{a}$	(95) 36.1${}^{b}$	(107) 30.4${}^{b}$
On Day 47±3	(38) 37.3${}^{\mathrm{ab}}$	(121) 45.0${}^{a}$	(31) 30.1${}^{b}$	(41) 27.2${}^{b}$	(71) 25.7${}^{b}$	(218) 45.2${}^{a}$	(67) 25.5${}^{b}$	(78) 22.2${}^{b}$
Pregnancy losses${}^{3}$	(5) 11.6${}^{b}$	(48) 28.4${}^{a}$	(9) 22.5${}^{\mathrm{ab}}$	(18) 30.5${}^{a}$	(13) 15.5${}^{b}$	(61) 21.9${}^{\mathrm{ab}}$	(28) 29.5${}^{a}$	(29) 27.1${}^{a}$

${}^{1}$ Cows showing estrus during the postpartum period received the CO-72 (EPG–PG) (GnRH+PGF${}_{2\alpha 1}+$PGF${}_{2\alpha 2}+$GnRH+TAI 48 h after PGF${}_{2\alpha 2}$) or ECO-72 (GnRH+PGF${}_{2\alpha }+$GnRH+TAI 72 h after PGF${}_{2\alpha }$). Cows that did not show estrus during the postpartum period received the CO-72 (NEPG–PG) (GnRH+PGF${}_{2\alpha 1}+$PGF${}_{2\alpha 2}+$GnRH+TAI 48 h after PGF${}_{2\alpha 2}$) or NECO-72 (GnRH+PGF${}_{2\alpha }+$GnRH & TAI 72 h after PGF${}_{2\alpha }$). ${}^{2}$ 22, 78, 64 and 73 cows from EPG–PG, NEPG–PG, ECO-72 and NECO-72 treatments, respectively, exhibited estrus and were inseminated before the last GnRH injection and excluded from the analysis. ${}^{3}$ Proportion of cows diagnosed pregnant at 33±3 d after AI that were diagnosed nonpregnant at 47±3 d after AI. ${}^{a,b}$ Percentages among treatments within a parity group with different superscripts differ (P≤0.05).

Overall pregnancy losses were similar in primiparous and multiparous cows. In primiparous cows, pregnancy losses were lower (P≤0.05) in the EPG–PG treatment than in both NEPG–PG and NECO-72 treatments with no differences between EPG–PG and ECO-72 treatments (Table 5). On the other hand, within multiparous cows, pregnancy losses were lower (P≤0.05) in the EPG–PG treatment than in both ECO-72 and NECO-72 treatments with no differences between EPG–PG and NEPG–PG treatments (Table 5). No interaction effect of treatment–parity was detected for pregnancy losses (P=0.40).

### 3.4 Effect of season and treatment–season interaction

Table 6 shows P/AI and PLs for cows that completed the hormonal protocols based on season and treatment. Pregnancy rates on days 33 and 47 post-TAI were detected between treatment and season. In hot and cold months, P/AI in cows receiving NEPG–PG protocol were higher (P≤0.05) than those in the cows receiving EPG–PG, ECO-72 and NECO-72 protocols on days 33 and 47.

**Table 6:** Pregnancies per AI and pregnancy losses for cows based on treatment–season interaction.

Parameter	Hot				Cold			
	Treatments${}^{1}$							
	EPG–PG	NEPG–PG	ECO-72	NECO-72	EPG–PG	NEPG–PG	ECO-72	NECO-72
	($n=71{)}^{2}$	(n=231)	(n=178)	(n=222)	(n=307)	(n=520)	(n=188)	(n=281)
Pregnancies per AI								
On Day 33±3	(21) 29.6${}^{b}$	(121) 52.4${}^{a}$	(74) 41.6${}^{b}$	(78) 35.1${}^{b}$	(106) 34.5${}^{b}$	(327) 62.9${}^{a}$	(61) 32.5${}^{b}$	(88) 31.3${}^{b}$
On Day 47±3	(19) 26.8${}^{b}$	(92) 39.8${}^{a}$	(42) 23.6${}^{b}$	(51) 23.0${}^{b}$	(90) 29.3${}^{b}$	(247) 47.5${}^{a}$	(56) 29.8${}^{b}$	(68) 24.2${}^{b}$
Pregnancy losses${}^{3}$	(2) 9.5${}^{c}$	(29) 24.0${}^{\mathrm{bc}}$	(32) 43.2${}^{a}$	(27) 34.6${}^{\mathrm{ab}}$	(16) 15.1${}^{\mathrm{bc}}$	(80) 24.5${}^{a}$	(5) 8.2${}^{c}$	(20) 22.7${}^{\mathrm{ab}}$

${}^{1}$ Cows showing estrus during the postpartum period received the CO-72 (EPG–PG) (GnRH+PGF${}_{2\alpha 1}+$PGF${}_{2\alpha 2}+$GnRH+TAI 48 h after PGF${}_{2\alpha 2}$) or ECO-72 (GnRH+PGF${}_{2\alpha }+$GnRH+TAI 72 h after PGF${}_{2\alpha }$). Cows that did not show estrus during the postpartum period received the CO-72 (NEPG–PG) (GnRH+PGF${}_{2\alpha 1}+$PGF${}_{2\alpha 2}+$GnRH+TAI 48 h after PGF${}_{2\alpha 2}$) or NECO-72 (GnRH+ PGF${}_{2\alpha }+$GnRH & TAI 72 h after PGF${}_{2\alpha }$). ${}^{2}$ 22, 78, 64 and 73 cows from EPG–PG, NEPG–PG, ECO-72 and NECO-72 treatments, respectively, exhibited estrus and were inseminated before the last GnRH injection and excluded from the analysis. ${}^{3}$ Proportion of cows diagnosed pregnant at 33±3 d after AI that were diagnosed nonpregnant at 47±3 d after AI. ${}^{a,b,c}$ Percentages among treatments within a season group with different superscripts differ (p≤0.05).

Overall pregnancy losses were higher in the hot months than in the cold months. In the hot months, PLs were similar between EPG–PG and NEPG–PG treatments, while both treatments were lower (P≤0.05) than the ECO-72 treatment. On the other hand, similar PLs were found for ECO-72 and NECO-72 treatments, while PL in the NECO-72 treatment was higher (P≤0.05) than in the EPG–PG treatment. In the cold months, PLs were similar for EPG–PG and ECO-72 treatments, while both treatments had lower PLs (P≤0.05) than the NEPG–PG treatment. On the other hand, similar PLs were found for EPG–PG and NECO-72 treatments, while PL was similar for NECO-72 and NEPG–PG treatments (Table 6).

### 3.5 Effect of parity–season interaction

A tendency (P≤0.1) for a parity–season interaction effect was found for pregnancy loss and P/AI on Day 47 but not on Day 33.

## 4 Discussion

Optimizing CL regression in specific groups of cows may be particularly challenging; hence, new alternatives should be explored to increase CL regression during the TAI protocols. The current study is the first to show the effect of administrating the luteolytic dose of PG in two injections on days 5 and 6 of the TAI protocol after of estrus expression comparing to the Cosynch protocol in a large dairy herd. This is the first study to demonstrate that the two PG injections can increase P/AI in different seasons without any P4 supplementation.

As in the previous study (Alnimer and Ababneh, 2014), the interval between the PG injection and the start of the treatment was an approach that simulated Presynch 14 protocol with one less PG injection and increased stage synchronization at the beginning of the protocols (Moreira et al., 2001; Vasconcelos et al., 1999). However, the interval from the setup injection of PG on Day 30 pp to the detection of estrus in the EPG–PG and ECO-72 groups complicated the procedures as cows showed estrus from as early as 2 d post-injection to as late as 7 d post-injection. Therefore, 12 d after estrus (day 0 of the cycle) was used in the current study and so the first GnRH injection was during diestrus around day 11 of the cycle. Due to the variation in the interval from PG to estrus, it was impossible to have a preplanned schedule for the injection or insemination before detection of estrus and injections were distributed on the weekdays. In contrast, cows in the NEPG–PG or NECO-72 had setup dates from the first PG injection on Day 30 pp.

In the present study, more cows (14.9 % and 12.7 %) in the CO-72 (ECO-72 and NECO-72) groups were observed in estrus prior to the last GnRH injection than cows (5.5 % and 9.4 %) in the PG–PG (EPG–PG and NEPG–PG) groups and were inseminated to maximize P/AI. These results were in line with (Santos et al., 2010), who reported that cows in CO-72 had larger ovulatory follicle diameters than cows in the 5 d protocols on the day of PG injection. We have previously reported that around 5 % to 10 % of cows showed estrus before the second GnRH in the TAI protocols (Alnimer and Ababneh, 2014; Alnimer et al., 2009, 2011), while 20 % of Ovsynch-treated cows displayed estrus within 48 h after PG as observed by DeJarnette et al. (2001). The stage of the estrous cycle at which a GnRH-based protocol is initiated affects the synchronization rate in different TAI protocols (Cartmill et al., 2001; Alnimer et al., 2009). On the other hand, a low percentage of 7.7 % (7.2 % to 8.2 %) in the CO-72 (ECO-72 and NECO-72) of cows showed estrus within a week after TAI than cows with 11.4 % (12 % and 10.7 %) in the PG–PG (EPG–PG and NEPG–PG) groups and were re-inseminated. These results agree with previous reports (Alnimer et al., 2009; Pursley et al., 1997; Puttabyatappa et al., 2018). The observation for estrus with other electronic detection systems should be used when applying timed insemination protocols. A lack of synchrony of estrus in the form of premature estrus or delayed estrus with fixed-time AI may significantly reduce P/AI. Therefore, for improved P/AI, cows with premature or delayed estruses should be inseminated when in estrus.

The proportion of cows that were in estrus was higher for cows that received two PG {EPG–PG} than for cows that received one PG {ECO-72} in this study. Luteolysis was greater for two PG injections compared to a single PG injection (Ribeiro et al., 2012). The two injections of PG on days 5 and 6 after the first GnRH were needed to maximize the percentage of cows that underwent complete CL regression before TAI. The group of cows that did not show heat (nonheat cow group) may be related to the P4 in the blood that we did not measure, as described by Fricke et al. (2016). They reported that low P4 cows were more likely to express estrus than high P4. This means that the nonheat cow group may have had a high level of P4 on day 30±3 pp. However, Santos et al. (2010) reported that cows that received two PG injections had less P4 concentrations compared with those that had one PG injection.

In the present study, the P/AI on day 47 was affected by treatment, parity and season, while PL was only affected by treatment and season. The results of this study demonstrated that P/AI at 47 pp for cows inseminated during and after VWP in the NEPG–PG treatment were higher than for cows in the EPG–PG, ECO-72 and NECO-72 treatments, respectively. Similar results in P/AI for cows treated with the CO-72 protocol in either heat or nonheat were found in previous study (Alnimer and Ababneh, 2014). These data are in agreement with (Santos et al., 2010), who reported that cows subjected to a 5 d protocol received two PG injections, whereas cows subjected to the 7 d protocol received only one PG injection, and cows receiving the 5 d protocol had more P/AI than cows receiving the 7 d protocol. Moreover, cows with the 5 d protocol required two injections of PG to achieve increased P/AI (Kasimanickam et al., 2009). Previously, Bridges et al. (2008) observed increased P/AI by reducing the interval from GnRH to PG from 7 to 5 d Thus, decreasing the interval between GnRH and the PG treatments from 7 d to 5 in the NEPG–PG increased P/AI. However, we are not sure why the nonestrus cow group had more P/AI. This group of cows may be cycling without showing heat due to the fact that first ovulation occurs within 3 weeks of calving in most dairy cows. Another potential underlying reason for the improved fertility observed with NECO-72 could be due to the high P4 level at the time of PG to allow for sufficient lysis of the CL. Recently, Wiltbank et al. (2015) reported that the second PG injection appeared to enhance fertility in cows with elevated P4 and not in cows with low P4. The two PG injections and P/AI may be related to an increase in the synchronization of ovulation near AI due to complete CL regression. A younger CL is difficult to regress with one PG injection (Santos et al., 2010; Ribeiro et al., 2012). In this way, we are reducing the duration of the development of the ovulatory follicle dominance and shortening the transition from follicular phase to luteal phase and induced ovulation to improve P/AI in our study. This is confirmed with our study, in which P/AI is higher with the two PG injections, especially the NE group through inducing cyclicity and increased stage synchronization of the cycle.

Regardless of treatment, the overall first-service P/AI were greater in primiparous cows than in multiparous cows. This finding is similar to the result of previous studies (Astiz and Fargas, 2013; Alnimer et al., 2009; Souza et al., 2008). Recently, Astiz and Fargas (2013) reported that a higher pregnancy rate was achieved in primiparous cows using Double Ovsynch synchronization than in multiparous cows. This is due to primiparous cows having a high sensitivity to the metabolic and endocrine signals during the pp period, such as those influenced by the nutrient balance (Santos et al., 2009). The anovulation that occurred in primiparous cows after pp may explain the higher P/AI on days 33 and 47 post-TAI.

The hot months resulted in significantly more PLs when compared to cold months. Our results agree with previous studies by Alnimer et al. (2009) and Hansen (2002), who reported that heat stress leads to a high PL rate. In the current study, overall P/AI were superior in cold months than in hot months. Similarly to the results of other workers who found greater P/AI in dairy cows during cold months compared with that during hot months (Stevenson et al., 2014; Alnimer et al., 2002). Heat stress has a negative effect on fertility in many ways, such as compromising steroidogenesis and oocyte quality, reducing the amount of P4 that is secreted by the CL and fertilization rate. High heat loads before or after AI lead to a disruption of oocyte maturation (Roth and Hansen, 2005), reduction of P/AI and affect the survival of the embryo after conception (Morton et al., 2007). Recently, De Rensis et al. (2017) reported that during the warm season there is an imbalance in the hypothalamic–pituitary–ovarian axis, which leads to a reduction in the reproductive performance of the cow and compromises the quality of oocytes.

The magnitude of embryonic loss in this study is not affected by parity. The EPG–PG tended to have a lower embryonic loss compared with other treatments. Our results agree with previous studies that reported greater PLs in anovular cows than in cycling cows (Cartmill et al., 2001; Cerri et al., 2004). Moreover, anovulatory cows are at risk in the establishment and maintenance of pregnancy (Santos et al., 2004).

## 5 Conclusion

Estrous response and luteal regression in dairy cows was improved with an additional treatment of PG administrated 1 d apart. Administration of two PG injections increased P/AI in lactating dairy cows subjected to the 5 and 6 d timed protocol. This study confirmed that a 2 d decrease in the period of follicle dominance (by reducing the interval between GnRH and PG in a TAI protocol) improves the fertility of lactating dairy cows. Furthermore, controlling temperature and looking for cooling alternatives in the summer might be more important than those of hormonal treatments.

## Data Availability

The data of the paper are available upon request from the corresponding author.
